# Association Between Obesity and Cardiovascular Outcomes: A Systematic Review and Meta-analysis of Mendelian Randomization Studies

**DOI:** 10.1001/jamanetworkopen.2018.3788

**Published:** 2018-11-16

**Authors:** Haris Riaz, Muhammad Shahzeb Khan, Tariq Jamal Siddiqi, Muhammad Shariq Usman, Nishant Shah, Amit Goyal, Sadiya S. Khan, Farouk Mookadam, Richard A. Krasuski, Haitham Ahmed

**Affiliations:** 1 Department of Cardiovascular Medicine, Cleveland Clinic, Cleveland, Ohio; 2 Department of Internal Medicine, John H. Stroger Jr Hospital of Cook County, Chicago, Illinois; 3 Department of Internal Medicine, Dow University of Health Sciences, Karachi, Pakistan; 4 Department of Preventive Medicine, Feinberg School of Medicine, Northwestern University, Chicago, Illinois; 5 Department of Cardiovascular Medicine, Feinberg School of Medicine, Northwestern University, Chicago, Illinois; 6 Department of Cardiovascular Medicine, Mayo Clinic, Phoenix, Arizona; 7 Department of Cardiovascular Medicine, Duke University, Durham, North Carolina

## Abstract

**Importance:**

Although dyslipidemia has been consistently shown to be associated with atherogenesis, an association between obesity and cardiovascular disease outcomes remains controversial. Mendelian randomization can minimize confounding if variables are randomly and equally distributed in the population of interest.

**Objective:**

To assess evidence from mendelian randomization studies to provide a less biased estimate of any association between obesity and cardiovascular outcomes.

**Data Sources:**

Systematic searches of MEDLINE and Scopus from database inception until January 2018, supplemented with manual searches of the included reference lists.

**Study Selection:**

Studies that used mendelian randomization methods to assess the association between any measure of obesity and the incidence of cardiovascular events and those that reported odds ratios (ORs) with 95% CIs estimated using an instrumental variable method were included. The 5 studies included in the final analysis were based on a consensus among 3 authors.

**Data Extraction and Synthesis:**

Two investigators independently extracted study characteristics using a standard form and pooled data using a random-effects model. The Meta-analysis of Observational Studies in Epidemiology (MOOSE) reporting guideline was followed.

**Main Outcomes and Measures:**

Obesity associated with type 2 diabetes, coronary artery disease, or stroke. The hypothesis was formulated prior to data collection.

**Results:**

Of 4660 potentially relevant articles, 2511 titles were screened. Seven studies were included in the systematic review, and 5 studies with 881 692 participants were eligible to be included in the meta-analysis. Pooled estimates revealed that obesity was significantly associated with an increased risk of type 2 diabetes (OR, 1.67; 95% CI, 1.30-2.14; *P* < .001; *I*^2^ = 93%) and coronary artery disease (OR, 1.20; 95% CI, 1.02-1.41; *P* = .03; *I*^2^ = 87%). No association between obesity and stroke was found (OR, 1.02; 95% CI, 0.95-1.09; *P* = .65; *I*^2^ = 0%).

**Conclusions and Relevance:**

The present meta-analysis suggests that obesity is associated with type 2 diabetes and coronary artery disease. Although this analysis of mendelian randomization studies does not prove causality, it is supportive of a causal association. Hence, health care practitioners should continue to emphasize weight reduction to combat coronary artery disease.

## Key Points

**Question:**

Do mendelian randomization data show that an association exists between obesity and cardiovascular outcomes?

**Findings:**

In this systematic review and meta-analysis of nearly 1 million participants, obesity was associated with type 2 diabetes and coronary artery disease but not with stroke.

**Meaning:**

Obesity may increase the risk of subsequent diabetes and may contribute to cardiovascular outcomes and should thus remain a major focus of public health initiatives.

## Teaser

This systematic review and meta-analysis evaluates evidence from mendelian randomization studies to provide a less biased estimate of any association between obesity and cardiometabolic outcomes, specifically coronary artery disease, stroke, or type 2 diabetes, among nearly 1 million participants.

## Introduction

Coronary artery disease (CAD) and its ensuing complications, including myocardial infarction and heart failure, continue to be the leading cause of morbidity and mortality in the developed world and increasingly so in the developing world.^1,2,3^ Dyslipidemia has consistently been shown to be associated with atherosclerosis. The mechanistic link between dyslipidemia and atherogenesis is supported by a large number of studies demonstrating a correlation between levels of low-density lipoprotein cholesterol and major adverse cardiovascular events.^4,5^ Further support is provided by studies with pharmacological agents, including statins, ezetimibe, and proprotein convertase subtilisin/kexin type 9 inhibitors, which reduce low-density lipoprotein cholesterol and improve cardiovascular disease (CVD) outcomes when used for either primary or secondary prevention.^6,7^

While the link between dyslipidemia and CVD is well established, an association between obesity and CVD remains controversial. This is important because the rising prevalence of obesity and metabolic syndrome may eventually offset the public health gains achieved by improved treatment of CAD.^8^ To complicate matters further, some investigators have proposed an “obesity paradox,” in which a higher body mass index (BMI) has been paradoxically associated with improved clinical outcomes.^9^ Observational studies are limited by bias and confounding variables, and generating randomized data is inherently challenging.

Mendelian randomization uses genetic variants to estimate the health consequences of phenotypes influenced by these genetic variants.^10^ It is a relatively novel epidemiologic study design incorporating genetic information into standard epidemiologic methods. Mendelian randomization offers an opportunity to study associations without many of the typical biases that are inherent in traditional epidemiologic approaches. Thus, mendelian randomization can fill the evidence gaps by minimizing confounding, if variables are randomly and equally distributed in the population of interest.^11^

To our knowledge, no study to date has pooled data from mendelian randomization studies. Individual mendelian randomization studies have yielded conflicting findings regarding the association between obesity and cardiometabolic outcomes. In an attempt to resolve these inconsistencies, we conducted a systematic review and meta-analysis of mendelian randomized studies to assess the existence and extent of any association between obesity and CVD.

## Methods

This systematic review and meta-analysis followed the Meta-analysis of Observational Studies in Epidemiology (MOOSE) reporting guideline and the American Heart Association guideline.^12,13^ The need for obtaining institutional review board approval or patient informed consent was waived for this study because it is a review of publicly available data.

### Data Sources and Search Strategy

MEDLINE and Scopus were searched from the inception of these databases to January 2018 by 2 independent researchers (H.R. and M.S.K.). Detailed search strategies for each database are given in eTable 1 in the Supplement. The reference lists of the retrieved articles and the relevant reviews were then screened to identify any pertinent studies.

### Study Selection

All articles retrieved from the systematic search were exported to EndNote reference library, version X8.1 (Clarivate Analytics), wherein duplicates were sought and removed. Two independent reviewers (H.R. and M.S.K.) assessed the remaining articles, and only those that met the predefined criteria were selected. A third investigator (T.J.S.) was consulted to resolve any discrepancies. Relevant articles were initially selected on the basis of the title and abstract, after which the full text was read to confirm relevance.

The following 2 eligibility criteria were used to select studies. The study conducted a mendelian analysis to assess the association between any measure of obesity (BMI [calculated as weight in kilograms divided by height in meters squared] or waist to hip ratio [WHR]) and cardiometabolic outcomes, and the reported results included odds ratios (ORs) with 95% CIs, which were estimated using an instrumental variable method.

### Data Extraction and Quality Assessment

We used both BMI and WHR adjusted for BMI (WHRadjBMI) as a measure of obesity for our systematic review. The ORs for the association between 1 SD increase in BMI and cardiometabolic outcomes were abstracted from all studies. For the present study, cardiometabolic traits included CAD, stroke, and type 2 diabetes (T2D). The following baseline and study characteristics were also extracted: sample size, mean age, number of single-nucleotide polymorphisms, measure of obesity used, database used, methods for determining BMI and WHRadjBMI, and outcome ascertainment.

Mendelian randomization rests on 3 main assumptions.^14^ Assumption 1 is that the genotype must be associated with the phenotype (here, obesity). Assumption 2 is that the genotype should not be associated with confounders. Assumption 3 is that the genotype should affect the outcome only through the risk factor. Although the first assumption can be easily evaluated, the second and third assumptions (collectively known as absence of pleiotropy) are hard to prove, and their evaluation rests mainly on the judgment of the investigators.^15^ Although several statistical tools have been recently proposed to verify these assumptions and protect against biasing of results through pleiotropic variants, these methods are generally inconclusive and may fail to identify bias in various circumstances.^16^ There are no standardized tools to ascertain the risk of bias in mendelian randomization studies while conducting a meta-analysis. Thus, to assess the quality of the included studies, we evaluated whether the 3 assumptions of the mendelian randomization were validated, and, if so, the method used for those validations.

### Statistical Analysis

Review Manager, version 5.3 (Copenhagen: The Nordic Cochrane Centre, The Cochrane Collaboration, 2014) was used to perform all statistical analyses. The ORs from individual studies were pooled using a random-effects model. Forest plots were created to visually assess the results of pooling. An outcome to be analyzed and reported in our quantitative analysis (meta-analysis) required a minimum of 2 studies reporting nonoverlapping data. When 2 or more studies reported data from the same source or databank, only the study with the most participants was included in the analysis. Studies not included in the meta-analysis were still included in the qualitative analysis (systematic review). The *I*^2^ statistic was used to assess heterogeneity across studies, with a value of *I*^2^ between 25% and 50% considered mild heterogeneity, between 50% and 75% considered moderate heterogeneity, and greater than 75% considered severe heterogeneity.^17^ Visual inspection of the funnel plot was performed to evaluate publication bias. A 2-sided *P* < .05 was considered statistically significant in all cases.

## Results

### Literature Search Results

**Figure 1.**
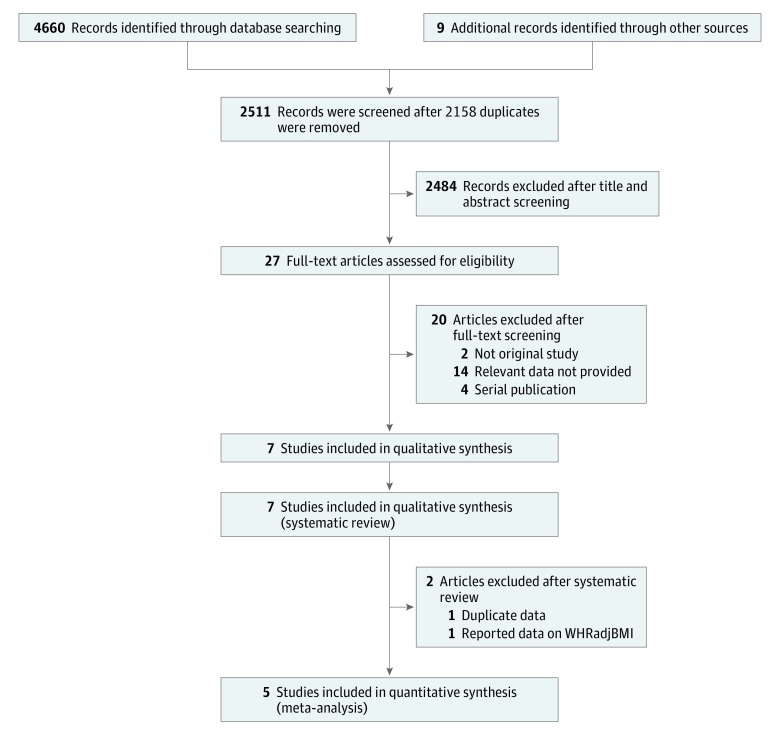
Flowchart Summarizing Results of the Literature Search WHRadjBMI indicates waist to hip ratio adjusted for body mass index calculated as weight in kilograms divided by height in meters squared.

An initial search of the 2 databases revealed 4660 potentially relevant articles, of which 2511 remained after excluding duplicates. After applying eligibility criteria, we selected 7 articles for inclusion in the systematic review.^18,19,20,21,22,23,24^ Those studies with overlapping data were excluded, leaving 5 articles for inclusion in the present meta-analysis.^18,19,21,23,24^ An analysis based on WHRadjBMI was not conducted in the present study because an insufficient number of studies used this measure. The Preferred Reporting Items for Systematic Reviews and Meta-analyses flowchart shown in Figure 1 summarizes the literature search.

### Study Characteristics and Quality Assessment

The 5 studies selected for quantitative analysis consisted of 881 692 participants. All included studies used BMI as a measure of obesity. The mean (range) age of the individuals in the pooled sample was 60 (50-64) years. These studies adjusted for a mean of 47 single-nucleotide polymorphisms (range, 9-97). Study characteristics of the included studies are given in eTable 2 in the Supplement. The definition of end points varied considerably across the different studies and may have led to higher statistical heterogeneity.

Visual inspection of the funnel plot suggested a low study bias (eFigure in the Supplement). The Table summarizes the validation of mendelian randomization assumptions by each study. Assumption 1 was validated in 4 studies, and assumptions 2 and 3 (absence of pleiotropy) were verified in 3 studies.

**Table.** Validation of the 3 Assumptions of Mendelian Randomization in Each Study

Source	Assumption 1^a^	Assumptions 2 and 3^b^	Conclusion
Nordestgaard et al,^21^ 2012	Strength of association between gene and BMI not estimated or reported from another study	No attempt was made to detect or adjust for pleiotropy.	None of the 3 assumptions validated
Fall et al,^24^ 2013	Association between gene and BMI not tested for; assumed to be sufficient based on previous studies	Pleiotropy could not be tested for statistically.	Only a single genotype was used as the instrument. Considerable risk of bias due to pleiotropy
Holmes et al,^18^ 2014	The *F* statistic was calculated to study the association between genes and BMI (*F* = 237).	Pleiotropy was not estimated.	Assumption 1 was validated. Pleiotropy was not tested for and is possibly present.
Hägg et al,^20^ 2015	Random-effects meta-analysis was used to test for association between genetic score and BMI. A strong association was found (*P* = 2.77 × 10^−107^).	Association of individual adiposity SNPs with CHD using CARDIoGRAMplusC4D data were investigated; this suggested that large pleiotropic effects were unlikely.	Assumption 1 valid. Pleiotropy not specifically tested for and could be present.
Lyall et al,^19^ 2017	*F* statistic calculated by the study was 2175.	MR-Egger analysis was conducted to detect and account for pleiotropy. The following covariates were used: Townsend deprivation index (*P* = .02), smoking status (*P* < .01), and alcohol intake (*P* < .001). These were adjusted for, and MR-Egger analysis did not suggest presence of unbalanced horizontal pleiotropy.	All 3 assumptions validated; pleiotropy was identified and adjusted for.
Dale et al,^23^ 2017	Association between genes and BMI not estimated	MR-Egger regression was broadly consistent with conventional MR analysis, showing little evidence of pleiotropy.	Assumption 1 not validated. Pleiotropy was likely minimal.
Emdin et al,^22^ 2017	Association between genes and WHRadjBMI not estimated in the study; *F* statistic reported from the UK Biobank was 1713.	Test for trend was performed across quartiles of the polygenic risk score for WHRadjBMI using logistic regression, with each potential confounder as the outcome. The association of the polygenic risk score with the following confounders was tested: smoking, alcohol use, physical activity, vegetable consumption, red meat consumption, and breastfeeding status as a child. No significant association was found. Five sensitivity analyses were also conducted, of which 4 were consistent with no pleiotropy.	Assumption 1 was considered valid based on data from the literature. Possible pleiotropy

Abbreviations: BMI, body mass index (calculated as weight in kilograms divided by height in meters squared); CARDIoGRAMplusC4D, Coronary Artery Disease Genome-Wide Replication and Meta-analysis plus the Coronary Artery Disease Genetics Consortium; CHD, coronary heart disease; DIAGRAM, Diabetes Genetics Replication and Meta-analysis; MR, mendelian randomization; SNP, single-nucleotide polymorphism; WHRadjBMI, waist to hip ratio adjusted for BMI.

^a^ Genotype must be associated with phenotype (obesity); validated in 4 studies.

^b^ Absence of pleiotropy (ie, genotype should not be associated with confounders and should affect outcome only through the risk factor); verified in 3 studies.

### Meta-analysis Results

**Figure 2.**
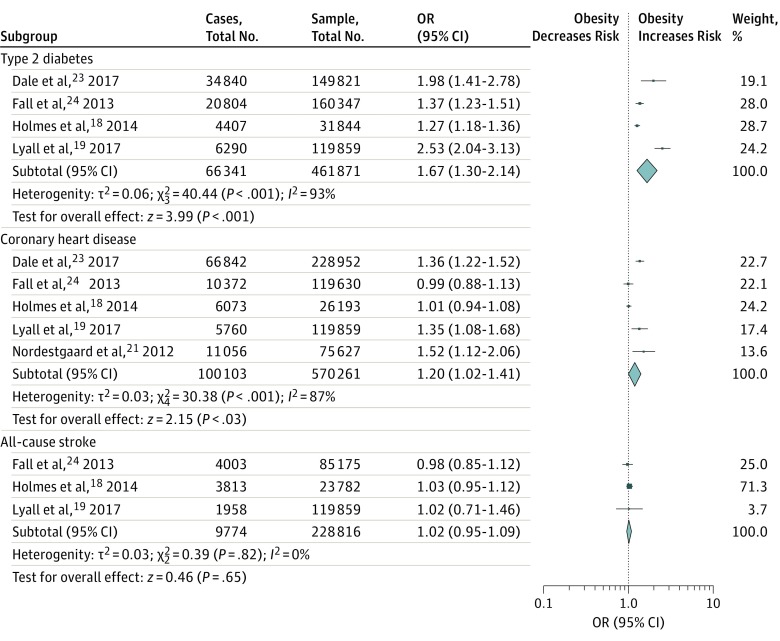
Meta-analysis Results Obesity has a statistically significant association with type 2 diabetes and with coronary artery disease but not with stroke. The size of the data markers indicates the weight of the odds ratio (OR), using random-effects analysis with instrumental variables.

The results of our meta-analysis are shown in Figure 2. Data on T2D were reported by 4 studies, with 461 871 participants. Our analysis showed a significant association between obesity and T2D (OR, 1.67; 95% CI, 1.30-2.14; *P* < .001; *I*^2^ = 93%). All included studies (n = 570 261 participants) reported CAD as an outcome. Obesity was significantly associated with CAD (OR, 1.20; 95% CI, 1.02-1.41; *P* = .03; *I*^2^ = 87%).

Three studies with 228 816 participants provided all-cause stroke as a clinical outcome. We found no significant association between obesity and all-cause stroke (OR, 1.02; 95% CI, 0.95-1.09; *P* = .65; *I*^2^ = 0%). An analysis that included only 180 795 patients with ischemic stroke showed no difference in the overall result (OR, 1.02; 95 CI%, 0.92-1.14; *P* = .65; *I*^2^ = 1%).

## Discussion

The results of the present study of nearly 1 million participants suggested an association between obesity and CAD. To our knowledge, this is the largest meta-analysis of obesity and CVD end points and the first to use mendelian randomization studies to pool findings. Each 1-SD increase in BMI increased the odds of T2D by 67% and of CAD by 20%. In light of the mendelian randomization, those increases are assumed to be independent of other traditional confounders.

The findings from this meta-analysis fill an important evidence gap and are timely for several reasons. First, obesity and the ensuing metabolic syndrome are becoming an epidemic, and a definitive association is necessary to inform decision making. If recent secular trends continue unabated, up to 20% of the world’s adult population (1.2 billion individuals) is expected to be obese by 2030, with the prevalence of metabolic syndromes such as diabetes and CVD to increase by 54% and 22%, respectively.^25,26,27^ Second, despite a number of efficacious therapies, patients at high risk of CVD or with a history of CVD have substantial residual risk, and it is important to identify factors responsible for this risk. For instance, compelling evidence exists regarding the efficacy of lipid-lowering agents in both primary and secondary prevention of CVD.^6,28,29^ However, despite such treatments and adherence to guideline-directed medical therapy, a substantial proportion of patients continue to experience cardiovascular events, underscoring the importance of the need to identify additional, novel risk factors. Third, although our study shows an association between obesity and CVD, this may not necessarily lead to increased mortality. Obesity may be associated with improved survival in patients with established CVD, a finding that is termed an “obesity paradox.”^30,31,32^ However, this concept is much debated because results from studies are inconsistent.^33,34^

Prior meta-analyses of observational studies on this subject have been limited by bias, such as that caused by smoking or confounding from prediagnostic weight loss associated with disease. Similarly, although some studies have attempted to adjust for confounding variables, such as diabetes, hypertension, and hypercholesterolemia, there has been concern regarding overadjustment. In addition, the substantial heterogeneity of the included studies (approximately 90% for CAD and diabetes) mandates that the results be interpreted with caution. This heterogeneity was anticipated, however, given the variation in study methods, participants, and localities.

Mendelian randomization assumes that the alleles of interest are randomly and equally distributed in the population of interest. For instance, genetic loci associated with obesity may be randomly (and equally) distributed in some people and not others. Comparing events of interest between people with the alleles of interest (and the ensuing higher BMI) and those without the alleles (and hence lower BMI) should therefore provide unbiased estimates between obesity and outcomes. We also assume that this method addresses several of the limitations conferred by traditional observational studies. For instance, although multivariate regression can be used to adjust for a number of confounding variables, the effect of smoking is so strong that conventional methods cannot sufficiently account for the association of smoking with BMI or with other outcomes of interest. By using mendelian randomization, it is assumed that any potential variables of interest are equally and randomly distributed in the population of interest, similar to a randomized controlled clinical trial.

### Limitations

Our analysis has several limitations. First, this is a pooled analysis of individual studies, and we did not have access to the individual patient data to conduct a patient-level analysis. Second, we studied obesity as a whole and could not perform a subgroup analysis of specific genetic mutations and their influence on the outcomes of interest. Third, although this analysis provides evidence for the harmful effects of obesity, we are unable to comment on the potential of interventions, such as lifestyle changes or pharmacological management, to attenuate any undesired effects. Fourth, we did not have an adequate number of studies to pool to assess the association of obesity with mortality (cardiovascular or all-cause). Fifth, this study assumed a linear association and therefore could not address the linearity (or lack thereof) of the underlying associations. Sixth, although mendelian randomization methods have several advantages over traditional meta-analysis methods and can provide evidence for an association between obesity and CVD, they remain modeling experiments and assumption dependent.

## Conclusions

Obesity was associated with an increased risk of T2D and CAD and should remain a major focus of public health initiatives. The present analysis of mendelian randomization studies was supportive of a causal association; however, it did not prove causality. Mendelian randomization assumptions were often not verified in individual studies, and this could have contributed bias to the present meta-analysis.

## Notes

Supplement. **eTable 1.** Search Strategy Used in Each Database Searched **eTable 2.** Baseline Characteristics of the Included Trials **eFigure.** Funnel Plot
