# Supplementary material for: Association Between Obesity and Cardiovascular Outcomes: A Systematic Review and Meta-analysis of Mendelian Randomization Studies
Source: JAMA Netw Open. 2018 Nov 16;1(7):e183788. doi: 10.1001/jamanetworkopen.2018.3788 (PMC6324374; doi:10.1001/jamanetworkopen.2018.3788)
Supplement: Supplement. — eTable 1. Search Strategy Used in Each Database Searched eTable 2. Baseline Characteristics of the Included Trials eFigure. Funnel Plot [file jamanetwopen-1-e183788-s001.pdf]

Supplementary Online Content

Riaz H, Khan MS, Siddiqi TJ, et al. Association between obesity and cardiovascular outcomes: a systematic review and meta-analysis of mendelian randomization studies. *JAMA Netw Open*. 2018;1(7):e183788. doi:10.1001/jamanetworkopen.2018.3788

eTable 1. Search Strategy Used in Each Database Searched

eTable 2. Baseline Characteristics of the Included Trials

eFigure. Funnel Plot

This supplementary material has been provided by the authors to give readers additional information about their work.

© 2018 Riaz H et al. *JAMA Network Open.*

eTable 1. Search Strategy Used in Each Database Searched

Database	Search Strategy	Articles retrieved
MEDLINE	(("obesity"[MeSH Terms] OR "obesity"[All Fields]) OR ("body mass index"[MeSH Terms] OR ("body"[All Fields] AND "mass"[All Fields] AND "index"[All Fields]) OR "body mass index"[All Fields]) OR ("body weight"[MeSH Terms] OR ("body"[All Fields] AND "weight"[All Fields]) OR "body weight"[All Fields]) OR ("adiposity"[MeSH Terms] OR "adiposity"[All Fields]) OR ("waist-hip ratio"[MeSH Terms] OR ("waist-hip"[All Fields] AND "ratio"[All Fields]) OR "waist-hip ratio"[All Fields] OR ("waist"[All Fields] AND "hip"[All Fields] AND "ratio"[All Fields]) OR "waist to hip ratio"[All Fields]) OR ("waist circumference"[MeSH Terms] OR ("waist"[All Fields] AND "circumference"[All Fields]) OR "waist circumference"[All Fields])) AND (("myocardial infarction"[MeSH Terms] OR ("myocardial"[All Fields] AND "infarction"[All Fields]) OR "myocardial infarction"[All Fields]) OR (acute[All Fields] AND ("coronary disease"[MeSH Terms] OR ("coronary"[All Fields] AND "disease"[All Fields]) OR "coronary disease"[All Fields])) OR (("coronary vessels"[MeSH Terms] OR ("coronary"[All Fields] AND "vessels"[All Fields]) OR "coronary vessels"[All Fields] OR ("coronary"[All Fields] AND "artery"[All Fields]) OR "coronary artery"[All Fields]) AND ("syndrome"[MeSH Terms] OR "syndrome"[All Fields])) OR ("coronary artery disease"[MeSH Terms] OR ("coronary"[All Fields] AND "artery"[All Fields] AND "disease"[All Fields]) OR "coronary artery disease"[All Fields]) OR ("coronary disease"[MeSH Terms] OR ("coronary"[All Fields] AND "disease"[All Fields]) OR "coronary disease"[All Fields] OR ("coronary"[All Fields] AND "heart"[All Fields] AND "disease"[All Fields]) OR "coronary heart disease"[All Fields]) OR ("ischaemic heart disease"[All Fields] OR "myocardial ischemia"[MeSH Terms] OR ("myocardial"[All Fields] AND "ischemia"[All Fields]) OR "myocardial ischemia"[All Fields] OR ("ischemic"[All Fields] AND "heart"[All Fields] AND "disease"[All Fields]) OR "ischemic heart disease"[All Fields] OR "coronary artery disease"[MeSH Terms] OR ("coronary"[All Fields] AND "artery"[All Fields] AND "disease"[All Fields]) OR "coronary artery disease"[All Fields] OR ("ischemic"[All Fields] AND "heart"[All Fields] AND "disease"[All Fields])) OR ("cardiovascular system"[MeSH Terms] OR ("cardiovascular"[All Fields] AND "system"[All Fields]) OR "cardiovascular system"[All Fields] OR "cardiovascular"[All Fields]) OR ("stroke"[MeSH Terms] OR "stroke"[All Fields]) OR (("heart"[MeSH Terms] OR "heart"[All Fields] OR "cardio"[All Fields]) AND metabolic[All Fields] AND traits[All Fields])) AND ((Mendelian[All Fields] AND ("random allocation"[MeSH Terms] OR ("random"[All Fields] AND "allocation"[All Fields]) OR "random allocation"[All Fields] OR "randomization"[All Fields])) OR (instrumental-variable[All Fields] AND ("analysis"[Subheading] OR "analysis"[All Fields])) OR (("genetic therapy"[MeSH Terms] OR ("genetic"[All Fields] AND "therapy"[All Fields]) OR "genetic therapy"[All Fields] OR "genetic"[All Fields]) AND ("association"[MeSH Terms] OR "association"[All Fields])) OR (causal[All Fields] AND ("association"[MeSH Terms] OR "association"[All Fields])))	1,753
Scopus	((TITLE-ABS-KEY (obesity) OR TITLE-ABS-KEY (body AND mass AND index) OR TITLE-ABS-KEY (body AND weight) OR TITLE-ABS-KEY (adiposity) OR TITLE-ABS-KEY (waist-to-hip AND ratio) OR TITLE-ABS-KEY (waist AND circumference))) AND ((TITLE-ABS-KEY (myocardial AND infarction) OR TITLE-ABS-KEY (acute AND coronary AND disease) OR TITLE-ABS-KEY (coronary AND artery AND syndrome) OR TITLE-ABS-KEY (coronary AND artery AND disease) OR TITLE-ABS-KEY (coronary AND heart AND disease) OR TITLE-ABS-KEY (ischemic AND heart AND disease) OR TITLE-ABS-KEY (stroke) OR TITLE-ABS-KEY (cardiometabolic AND trait))) AND ((TITLE-ABS-KEY (mendelian AND randomization) OR TITLE-ABS-KEY (instrumental-variable AND analysis) OR TITLE-ABS-KEY (genetic AND association) OR TITLE-ABS-KEY (causal AND association)))	2,907

© 2018 Riaz H et al. *JAMA Network Open.*

eTable 2. Baseline Characteristics of the Included Trials

Studies	Measure of obesity	Mean, (SD) BMI/ WHRadjBMI, kg/m2	Total sample	Age, mean y	Men (%)	Number of SNPs used	Database	Cardio-metabolic Outcomes				Definition of endpoints	Ascertainment of BMI/WHRadjBMI
								CAD, (%)	Heart Failure, (%)	T2DM, (%)	Stroke, (%)		
Lyall, 2017	BMI	27.4(4.8)	119,859	57	47	93	UK Biobank (2006-2010)	5760 (4.8)	N/A	6290 (5.3)	1958 (1.6)	CAD defined as either angina or myocardial infarction	Body mass index was derived from weight in kilograms divided by height in meters squared
Dale, 2017	BMI / WHRadjBMI	BMI: 27.4(4.6)<br><br>WHRadjBMI: 0.89 (0.13)	213,556	64	N/A	BMI: 97<br><br>WHRadjBMI: 49	14 prospective studies, CAD from CARDIoGRAMplusC4D, Ischemic Stroke from METASTROKE, T2DM from DIAGRAM	66842 (29.1)	N/A	34840 (23.3)	N/A	UCLEB studies defined CHD as fatal or non-fatal myocardial infarction, or a coronary revascularization procedure, but excluding angina. CARDIoGRAMplusC4D used standard criteria for defining cases of CAD T2Dm definition follows DIAGRAM.	waist circumference, hip circumference and weight were used to calculate BMI and WHRadjBMI.

© 2018 Riaz H et al. *JAMA Network Open.*

eTable 2. Baseline Characteristics of the Included Trials (Continued)

Studies	Measure of obesity	Mean, (SD) BMI/ WHRadjBMI, kg/m2	Total sample	Age, mean y	Men (%)	Number of SNPs used	Database	Cardio-metabolic Outcomes				Definition of endpoints	Ascertainment of BMI/WHRadjBMI
Emdin, 2017	WHRadjBMI	27.5 (4.8)	768,269	57	48	48	Summary level data from 6 GWAS consortia (2007-2015), DIAGRA, CARDIoGRAMplusC4D, UK Biobanks (2007-2011)	66440 (22.4)	586 (0.52)	40530 (15.5)	2035 (1.8)	(International Statistical Classification of Diseases and Related Health Problems, Tenth Revision codes I21-I23).	WHR adjusted for BMI was derived in the UK Biobank through inverse normal transformation of WHR after adjustment for age, sex, and BMI.
Hagg, 2015	BMI	25.9 (4.5)	22,193	50	N/A	32	9 prospective cohorts within the ENGAGE Consortium, CARDIoGRAMplusC4D	3062 (13.8)	N/A	N/A	N/A	Pre-specified criteria set by each study	Body mass index was derived from weight in kilograms divided by height in meters squared

© 2018 Riaz H et al. *JAMA Network Open.*

eTable 2. Baseline Characteristics of the Included Trials (Continued)

Studies	Measure of obesity	Mean, (SD) BMI/ WHRadjBMI, kg/m2	Total sample	Age, mean y	Men (%)	Number of SNPs used	Database	Cardio-metabolic Outcomes				Definition of endpoints	Ascertainment of BMI/WHRadjBMI
Holmes, 2014	BMI	27.5(1)	34,538	60	35	14	8 cohorts that been genotyped by the Human CVD BeadArray	6073 (17.6)	N/A	4407 (13.5)	3813 (12.5)	Pre-specified criteria set by each study	Body mass index was derived from weight in kilograms divided by height in meters squared
Nordestgaard, 2012	BMI	N/A	75,627	N/A	N/A	9	Two population based and one-case-control study in Copenhagen	11,056	N/A	N/A	N/A	World Health Organization International Classification of Diseases: ICD8 410–414; ICD10 I20–I25)	Body mass index was derived from weight in kilograms divided by height in meters squared
Fall, 2013	BMI	N/A	198,502	N/A	N/A	24	36 population-based studies of individuals of European descent	10,372	6,068	20,804	4,003	Pre-specified criteria set by each study	Body mass index was derived from weight in kilograms divided by height in meters squared

Abbreviations: BMI: body mass index; WHRadjBMI: waist-hip ratio adjusted for BMI; CARDIOGRAMplusC4D: Coronary Artery Disease Genome-Wide Replication and Meta-analysis plus the Coronary Artery Disease Genetics Consortium; DIAGRAM: Diabetes Genetics Replication and Meta-analysis; SNP: single-nucleotide polymorphism; CHD: coronary heart disease; MR: mendelian randomization; N/A: not available; SD: standard deviation; CAD: coronary artery disease; T2DM: type 2 diabetes mellitus; ICD: international classification of diseases; GWAS: genome-wide association studies.

© 2018 Riaz H et al. *JAMA Network Open.*

eFigure. Funnel Plot

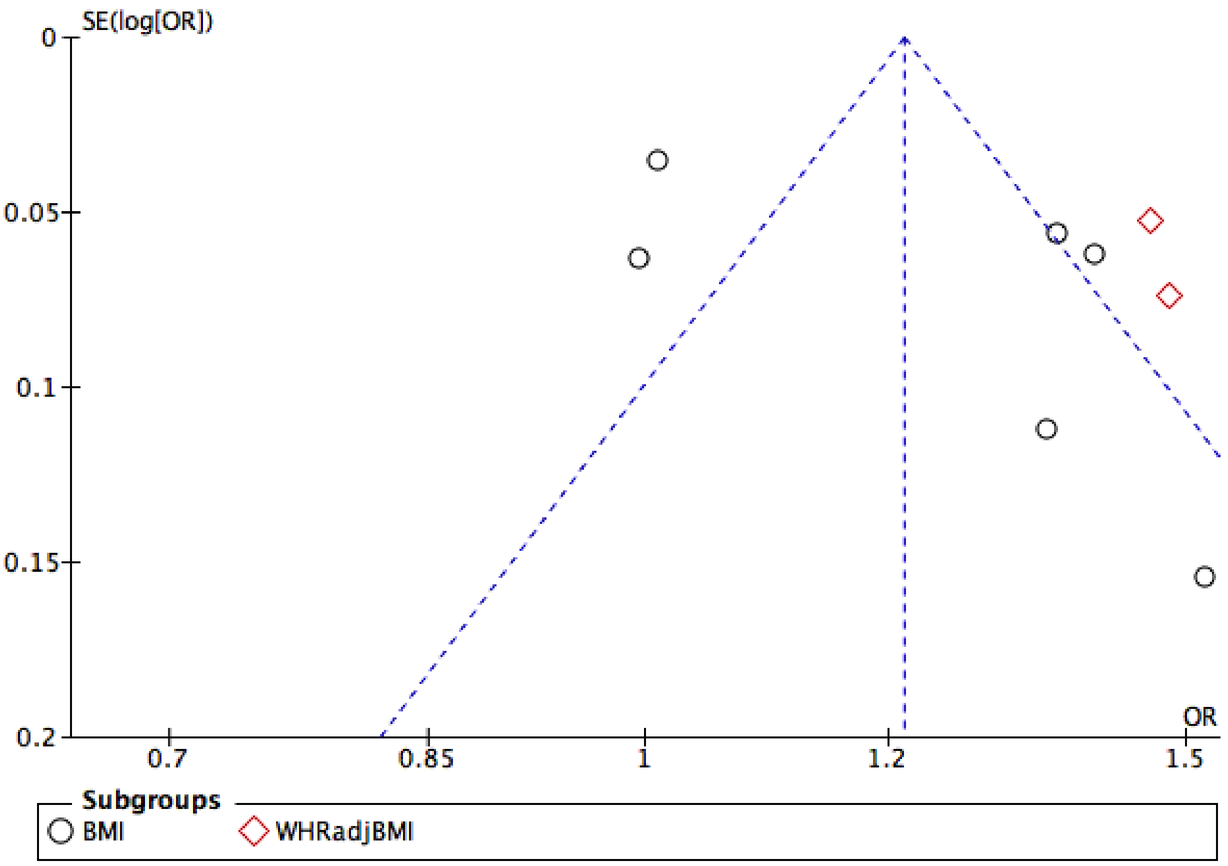

© 2018 Riaz H et al. *JAMA Network Open.*
